# Health Tract, No. 283

**Published:** 1867-03

**Authors:** 


### THE LAST HOPE.

Henry Clay and Andrew Jackson, and a long list of the celebrities of a former generation, after they had attained the highest positions possible to them, seem to have found that they did not bring that quiet, enduring and satisfying happiness which they were supposed to have possessd, and turned their disappointed eyes and hopes at last to membership in the Christian Church and the communion with saints, as the only pillow of repose on which to rest their weary heads, after the stormy conflicts of life had ended.

Within a few months, two ex-presidents and several military and veteran public servants, have sought by obtaining admission to the communion table to soothe life's sorrows and disappointments in the contemplation of the eternal mysteries which lie beyond the bounderies of Time ; for as men grow old they find themselves more and more inclined to look to the religion of the Bible as the only thing not entitled to be numbered among the vanities of the world. With still greater interest do those who have been cradled in the Church from early youth, desire to look for happiness in the hopes, and comforts, and promises of the Bible, as they near the confines of eternity ; as they grow older, and their steps become more tottering. and their vision dims, and their memory fails, and they are more and more left alone in the world, by the steady and rapid inroads which death makes in the list of early friends, associates and kindred, they turn their failing spirits to the Bible, and more eagerly bend their weary steps to the sanctuary, to have their hopes brightened and their faith stayed in the expositions of the scriptures. To them the "Law of the Lord" is an increasing delight, and every Sabbath they learn more and more of the truthfulness of the sentiment enunciated by the sweet singer of Israel, " A day in thy courts is better than a thousand anywhere else ; and that to be a doorkeeper in the house of God where one can at least stand on the outside and be in hearing of the songs of the sa ints is better than to serve in the palaces of Kings."

Many old persons go to church who cannot hear a word, as if they wished at least to be found in the way of duty, and be in the Savior's shadow, should he chance to pass along. It is sad, but in a sense a pleasant sight sometimes, to watch deaf persons in church, how earnestly they look at the minister, and with spread palm behind the ear, they seem to want to catch at least a falling crumb to appropriate to their christian needs, for aliment to the hungry soul ; and we can not think of any sweeter charity, next to that of making up a fund, the interest of which would generously provide for the reasonable wants of worn out and invalid ministers, and their helpless widows and orphan chtldren, than to have all the seats in our churches just fronting the pulpit, free to the old ; and furnished with an apparatus now enjoyed only by the wealthy few, which would enable the deaf to hear every word uttered by the minister ; then, indeed, would "The deaf hear, and the poor have the gospel preached unto them."

